# Blood groups and human groups: Collecting and calibrating genetic data after World War Two

**DOI:** 10.1016/j.shpsc.2014.05.008

**Published:** 2014-09

**Authors:** Jenny Bangham

**Affiliations:** Max Planck Institute for the History of Science, Boltzmannstr. 22, 14195 Berlin, Germany

**Keywords:** Blood groups, Genetics, Populations, Collection, World Health Organization, National Blood Transfusion Service

## Abstract

Arthur Mourant's *The Distribution of the Human Blood Groups* (1954) was an “indispensable” reference book on the “anthropology of blood groups” containing a vast collection of human genetic data. It was based on the results of blood-grouping tests carried out on half-a-million people and drew together studies on diverse populations around the world: from rural communities, to religious exiles, to volunteer transfusion donors. This paper pieces together sequential stages in the production of a small fraction of the blood-group data in Mourant's book, to examine how he and his colleagues made genetic data from people. Using sources from several collecting projects, I follow how blood was encountered, how it was inscribed, and how it was turned into a laboratory resource. I trace Mourant's analytical and representational strategies to make blood groups both credibly ‘genetic’ and understood as relevant to human ancestry, race and history. In this story, ‘populations’ were not simply given, but were produced through public health, colonial and post-colonial institutions, and by the labour and expertise of subjects, assistants and mediators. Genetic data were not self-evidently ‘biological’, but were shaped by existing historical and geographical identities, by political relationships, and by notions of kinship and belonging.

## Highlights

• Arthur Mourant's *The Distribution of the Human Blood Groups* (1954) was a vast collection of population genetic data.
• It presented blood-group frequencies as a ‘scientific’ methodology for studying human diversity.
• The paper follows how Mourant and his colleagues made population genetic data.
• Data were structured by the ways in which populations were defined and accessed by researchers.
• Populations were shaped by institutions, governments, and historical, racial and national identities.

When citing this paper, please use the full journal title *Studies in History and Philosophy of Biological and Biomedical Sciences*

## 1 Introduction

In 1954, British haematologist Arthur Mourant finished a book that constituted the largest single collection of human genetic data ever published: *The Distribution of the Human Blood Groups*. Mourant, a physician, serologist and geneticist, was director of the Blood Group Reference Laboratory in London, an institution internationally recognized as a centre for expertise on blood grouping techniques. During the previous few years, Mourant had carved out a successful research programme collecting data on the blood-group frequencies of people around the world. Blood groups were almost the only human traits with clear-cut Mendelian inheritance, and for Mourant and many others, blood-group genetics offered a promising set of methods for the study of racial diversity. Published by Blackwell Scientific Publications in Oxford, *The Distribution of the Human Blood Groups* was 400 pages long and contained the results of tests done on half-a-million people represented in nine maps, 40 tables and a vast bibliography. The *American Journal of Physical Anthropology* described Mourant's book as “indispensible”, the *American Anthropologist* called it “brilliant”, and the Royal Anthropological Institute journal *Man* considered it to be “the most important” contribution to “the anthropology of blood groups” to date (Birdsell, 1956, Boyd, 1955, Kherumian, 1954). The book's opening page explained Mourant's confidence in what blood groups could offer the study of race:

[a] study of the blood groups besides having many purely scientific advantages over most other bases of classification has the merit of providing objective criteria far removed from the traditional marks of ‘race’. We may plausibly though wrongly hold that fair or dark hair is the nobler; we may consider that a long face or a small foot is a mark of aristocracy—but the blood groups have so far remained almost completely free from the effects of such subjective judgements. (Mourant, 1954, p. 1)

**Fig. 1:**
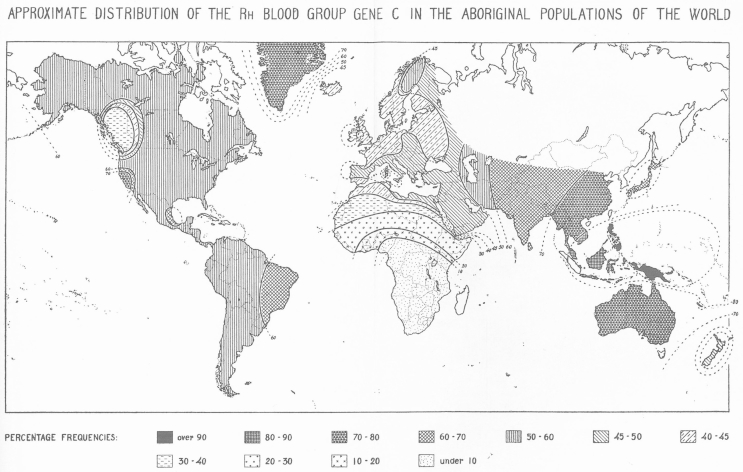
One of nine fold-out maps in *The Distributions of the Human Blood Groups* (1954). It shows the percentage of individuals carrying blood-group allele *C* in different geographical regions of the world, and uses isolines and shading to indicate threshold frequencies across space. This obscures evidence of the patchiness of sampling, the circumscription of geographical boundaries, and the political borders that structured collections. Permission to reproduce the image could not be obtained because the copyright holder, Blackwell Scientific Publications Oxford, no longer exists. The image is reproduced under provisions of ‘fair dealing’ for purposes of research, criticism, and review.

The objectivity of blood groups was rendered visually in the foldout maps at the back of the book (example in Fig. 1). On these maps, superimposed on an outline of the world's countries, isolines indicate threshold blood-group frequencies and the density of shading depicts their magnitude. These graphical techniques offer the impression of a smooth diffusion of blood-group alleles across geographical space, and obscure the different kinds of population represented in the data. It is one of the main purposes of this paper to reflect on the construction of these populations and to follow the social, political and institutional relationships that shaped Mourant's genetic data.

For many decades blood groups had been used to articulate multiple political discourses. Briefly, that history begins in 1901 when immunologist Karl Landsteiner observed that mixing samples of blood from different individuals sometimes caused red cells to clump together, or ‘agglutinate’. Landsteiner accounted for patterns of agglutination by categorizing people into groups, eventually standardized to A, B, O and AB. Landsteiner understood agglutination to be a simple immunological reaction: soluble ‘antibodies’ (anti-A, anti-B) in the serum of one sample reacting with ‘antigens’ (A, B) on the red cells of the other. Blood groups were simultaneously antigens and categories accounting for agglutination patterns. Their rising significance in transfusion medicine after the First World War led to a vast proliferation of studies on the blood-group frequencies of different racial and national populations, which were mobilized to serve post-First World War discourses on nationalism, colonialism, race and ancestry.^1^ By 1939 research on the geographical distributions of blood groups had involved tests on an estimated 1.3 million people.^2^ No less political, in Britain especially, blood groups were promoted as Mendelian traits, emblematic of a ‘reformed’ and ‘quantitative’ human heredity (Bangham, 2013a, Mazumdar, 1992). By the postwar period, the notion that blood-group genetics exemplified a modern, ‘scientific’ and ‘objective’ method for studying human diversity was perfectly in line with larger-scale postwar arguments about genetics and the purification of race science, elevated onto the international stage most publically by UNESCO (Brattain, 2007, Gormley, 2009, Reardon, 2004). By the 1950s, blood groups were seen by many as *the* pre-eminent traits for the study of human diversity, and Mourant had established himself as a worldwide expert on their study.

Mourant's success was closely associated with his institutional positions (Misson, Bishop, & Watkins, 1999; Mourant, 1995). After the Second World War he took charge of the Blood Group Reference Laboratory (BGRL), which was responsible for producing standardized reagents (‘antisera’) for blood-grouping tests in transfusion depots across Britain. In 1950 the World Health Organization (WHO) sanctioned the BGRL as its centre for antisera production. This appointment placed Mourant at the centre of a large international community of blood grouping laboratories. He provided reagents and technical advice to transfusion specialists, doctors, missionaries, geneticists and anthropologists around the world. Mourant established networks for extracting, labelling and moving blood from the field to the laboratory, networks that were later consequential for the protagonists of some of the other essays in this issue (especially those by Radin and Suárez-Díaz).^3^ Mourant himself proudly recounted how his congenial international working relationships put him in a “unique position” for collecting “specimens of blood for anthropological work”.^4^ So successful was Mourant at acquiring blood samples and records that he soon established a whole new centre for collating, organizing and analysing results: the Nuffield Blood Group Centre on the premises of London's Royal Anthropological Institute. There, Mourant oversaw a clerk, librarian and statistician responsible for the work of turning blood-group results into genetic data.

Building on existing accounts of how blood groups were mobilized to articulate broader political agendas,^5^ it is the argument of my paper that prevailing knowledge about human history, race and nation were also incorporated into the practices that produced blood-group data.^6^ Section two considers how researchers decided on which groups of people were appropriate and accessible for study, and asks how institutional and political conditions shaped those decisions. Section three deals with the people who collected blood samples and asks whose bodies were chosen for research. Section four deals with the practices through which transfusion records were obtained from clinical settings, and how researchers produced data from those records.^7^ Section five focuses on the paper work that took place at the Nuffield Blood Group Centre and outlines how the collation and aggregation of data might have shaped its meanings. The last section looks more closely at Mourant's data in *The Distribution of the Human Blood Groups*, and reflects on how its analysis and representation made it both credibly ‘genetic’ and understood as relevant to human ancestry, race and history.

## 2 Fieldwork destinations

How did researchers decide which populations to sample? Mourant's first research project on blood-group distributions began with a puzzle that geneticists had raised in the early 1940s. Soon after the Rhesus blood groups had been discovered, they were found to cause an incompatibility between Rhesus-negative women and their Rhesus-positive foetuses, which could result in life-threatening anaemia in newborns. Population geneticist J. B. S. Haldane and serologist Alexander Wiener had independently pointed out that the lethal consequences of this condition should have caused the rarer of the Rhesus alleles (in this case, the Rhesus-negative allele) to be selected out of populations (Haldane, 1942, Wiener, 1942). Haldane had suggested that the existence of this ‘polymorphism’ might be explained if modern Europeans were derived from the mixing of two ancestral groups, one carrying only Rhesus-positive, and the other only Rhesus-negative, alleles.^8^ Mourant set out to test the idea that somewhere in Europe the descendants of this hypothetical Rhesus-negative population still existed with a higher-than-normal frequency of Rhesus-negative alleles. But to find evidence of this ‘ancestral’ population he did not carry out a systematic survey of blood samples across Europe. Instead, Mourant started with what looked like the most likely candidates: the Basques.

Why was this population so compelling? The Basques had served a multiplicity of historical narratives about human history. One researcher sarcastically pointed out that the Basques, at one time or another, had been supposed akin to the “ancient Egyptians, Guanches, Berbers, Etruscans, Phoenicians, Lapps, Finns, Bulgarians, or to Asiatic races”, even “the sole survivors of Atlantis!”.^9^ In the mid-twentieth century it was commonplace that the Basque population was a Palaeolithic ‘relic’. Particularly striking to anthropologists, historians and linguists were the local language (“many Basques still speak the same language as in the Stone Age”), and head shape (modern Basques resembled “human remains in the Neolithic dolmens of the Basque country”).^10^ The Basques were “interesting relic[s] of Iberian times”, and a “perfectly definite ethnic group, both in their racial characters and in their traditional culture”.^11^

A prominent Basque ethnographer and Catholic priest living in London, José Miguel de Barandiarán, pointed to the scientific importance of the population in an appeal in the *Journal of the Royal Anthropological Institute* (*JRAI*) promoting scientific research on this “special race without analogy with any other known group”. de Barandiarán belonged to a community of Basque exiles living in London and Paris owing to persecution from Franco's regime. In his article de Barandiarán suggested that through their research, anthropologists might help persuade governments to “respect and protect the ethnic elements of the Basque people, not only for [their] antiquity, but [also] for [their] scientific interest” (de Barandiarán, 1946, p. 96). It was de Barandiarán who helped Mourant access Basques “of unmixed descent” for his first round of blood sampling in London and Paris (Chalmers, Ikin, & Mourant, 1948).

Elsewhere researchers accessed populations through doctors and public health facilities. Mourant used his vast network of correspondents to offer institutional and personal connections, reagents, and blood-grouping services to researchers on collecting expeditions to different parts of the world. A young Oxford medical student called Anthony Allison orchestrated two such expeditions in the late 1940s, one to Kenya and one to northern Scandinavia.^12^ In 1949, Allison had recently completed his doctorate when the Oxford University Exploration Club (OUEC) proposed a botanical and ornithological expedition to Mount Kenya in the summer vacation.^13^ Allison applied to join them and test the blood groups of local people. As a student he had attended lectures by R. A. Fisher, who had inspired him to work on human populations and who had given him a letter of introduction to Mourant. Allison went to the BGRL to be trained in blood-grouping tests and Mourant gave him antisera for the expedition. Building on the success of the Kenya trip, the following year Allison planned an expedition to Northern Scandinavia to test the blood groups of the ‘Lapps’.^14^

Two overlapping aspects of the colonial setting in Kenya were relevant in making it the destination of Allison's first expedition. First, he had spent his childhood there, and had developed a strong interest in the natural history and anthropology of the area (Carroll, 2008). Second, Kenya was under British colonial rule, and the OUEC, which funded two to three expeditions per year, had already organized two expeditions to the country since the end of the war. Affirming the importance of Kenya as a scientific destination for British researchers, over half the money for the expedition was granted by the Colonial Research Committee, which had been established in 1940 as a part of a large expansion of scientific research in the British colonies.^15^ The Committee's objective was to “rationalize the development process” using scientific research (Clarke, 2007, p. 479). A blood group survey would have fitted nicely with the Committee's stated goal of supporting the scientific study of colonial environments and societies, seen as a precursor for schemes such as disease control.^16^

For access to populations and apparatus, Allison relied upon local networks of medics and public health workers. Recalling the planning of expeditions, Allison explains that it was important first and foremost to obtain introductions to medical doctors, “beginning on a national level and extending to the local level”.^17^ For his expedition to Northern Scandinavia, Mourant gave Allison introductions to workers in the Oslo, Stockholm and Helsinki Blood Transfusion Laboratories, who carried out most of the blood-grouping tests and introduced Allison to local medical doctors. In turn, those doctors introduced Allison to Sami individuals with medical qualifications who would accompany him to local villages, hospitals and schools, also testifying to the significance of local institutions for defining samples. In Kenya too, Allison used a branching network of contacts: the Director of the Medical Research Laboratory in Nairobi was family friend, and through him Allison elicited introductions to District Medical Officers, and local medical assistants who could accompany Allison on his collection forays. Thus colonial administrative networks and institutions helped to shape decisions about which national, tribal, enclave and social groups were accessible and appropriate to study.

Colonial states routinized and reified racial categories through census practices, patterns of employment and land appropriation, the establishment of ‘native reserves’, and educational and medical services (Tilley, 2011, p. 219). The kinds of populations that geneticists and anthropologists tended to study—‘isolated’, ‘ancestral’, ‘unmixed’—were often precisely those subjected to the greatest colonial or other social administrative control. Just as Allison recruited many of his participants in medical centres, it is easy to conjecture how other bureaucratic and institutional structures shaped the sites of blood collection, both in Kenya—under British rule until 1963—and among the Sami, who were subject to Swedish, Norwegian and Finnish laws.

In short, colonial administrative structures and international health institutions shaped the constitution of specimen collections. Mourant's WHO networks were also important, as his Reference Laboratory provided help to researchers on expeditions, including offering to do blood-grouping tests, giving advice on how to collect and transport blood, and effecting introductions to people with equipment and specific expertise.

## 3 Making samples

Mourant wrote detailed instructions to anthropologists on the technical procedures of taking blood.^18^ But almost absent from his papers, books and private correspondence were reflections on how to decide which *individuals* to take blood from. This was despite the fact that Mourant and other researchers often sampled from communities that were geographically displaced from their place of ancestral identity, and that Mourant himself encountered some difficulties during his early Basque studies.

He made his earliest blood collections from Basque exiles in London and Paris, and augmented those with samples sent by a doctor in San Sebastian known to de Barandiarán. For all three sites, Mourant asked collectors to select participants based on their “personal names”, which, he said, incorporated “the names of several generations of ancestors” (Chalmers, Ikin, & Mourant, 1949, p. 351). But testing the samples Mourant was disappointed to find that they yielded a smaller proportion of Rhesus-negative individuals than he had hoped. 25.8% was higher than the recorded 16% average for the rest of Europe, but was not as high as the Rhesus-negative frequency recently recorded by blood-transfusion workers among Basques in Argentina. Convinced that the percentage must be higher, he resolved to obtain what he called a more “representative” sample, and this time his colleague Marshall Chalmers travelled to the South-West of France to collect “a larger number of specimens under expert anthropological guidance”. This “guidance” came once again came from de Barandiarán, who “assured” Chalmers of the “family relationships” and “racial purity” of each person tested (Chalmers et al., 1949, p. 531). Even after Chalmers had made his collection, the researchers further sifted the specimens by “eliminating the small number of persons who were believed to be of mixed race, and the few others who were blood relations of other persons tested” (Chalmers et al., 1949, p. 532). Affirming purity was clearly important, but the paper did not elaborate on what guided Chalmers in his belief over who was “mixed” and who was “Basque”.

Based on the calculated Rhesus frequencies, the researchers judged that this second round of collecting had indeed produced 383 samples from “the ‘purest’ available Basques”, and that these had yielded a Rhesus-negative frequency of 29%, apparently satisfactorily higher than the rest of Europe.^19^ The circularity of this reasoning notwithstanding, they cited this as good supporting evidence for the hypothesis that the Basques represented an original, pure Rhesus-negative population that had slowly been mixing with the Rhesus-positive peoples of the rest of Europe: “the Basques, while they may be akin to the Celtic and other peoples of the fringes of Europe, have retained a racial purity” not found elsewhere (Chalmers et al., 1949, p. 530). Reporting the story, the US *Science News-Letter* extended this conclusion further to declare that the Basques were an “almost pure representative race of ancient Europe” (‘Rh Factor Clue to Race’, 1948).

That researchers insisted on the ‘racial purity’ of an individual before taking their blood points to one way in which collection protocols configured published results. Another way was the decision to put sampling choices in the hands of a local trusted expert, a practice used across study sites. In Scandinavia, Allison employed “local medical assistants” to help him find appropriate and willing participants. These assistants knew “who were purebred Lapps and who were half-breeds from their names, the languages they spoke and enquiries about parents and grandparents”. In Kenya, Allison's local assistants “knew their fellow tribespeople from those belonging to other tribes” and checked the identities of “parents and grandparents” of participants.^20^ And just as Mourant and Chalmers drew on the expertise of de Barandiarán, over in Oregon, US anthropologist and serologist William Laughlin trusted a Basque lawyer to help him with an intensive survey of the blood groups of the local Basque community. Anthony Yturri, described by Laughlin as a “Basque university graduate”, offered to “contact two hundred Basque speaking Basques, with intact lineages”.^21^ Sampling in Oregon relied on Yturri's enquiries about names, grandparents and language, and his expert judgement.

Local medical assistants also helped persuade individuals to give their blood to researchers. Although the quantities taken for grouping tests were small, the procedure was potentially intrusive. Mourant recommended two alternative extraction methods, the easiest and cheapest being to prick an earlobe or finger and collect a few drops of blood in a specially prepared glucose and citrate solution. Mourant's preferred method, however, was the more sterile (and dramatic) ‘venepuncture’ technique. Applying a tourniquet to the arm, the doctor would use a fresh Bayer's ‘venule’—an automatic combined syringe and sample tube—to withdraw 2–5 cc of blood directly into a tube, which was then sealed. Doctors in Britain used this method in routine blood collection, and Mourant recommended that only those medically qualified should use it in field conditions. He strongly encouraged serologists to invest in expensive Bayer venules because they were particularly good at keeping specimens fresh, and because it was “even more expensive to pay the cost of airfreight on specimens which are unfit for testing”.^22^ Whichever technique was used, the collectors packed the samples on ice to keep them at a temperature just above 0°C. Allison recalled that blood collections were followed by a rush to Nairobi airport to get the ice-packed venules flown to London.^23^

Published reports disclose few instances of resistance by would-be research subjects, but it is clear that collectors could not always take participation for granted. Allison himself noted in his published work when populations were “un-cooperative” (Allison, Ikin, & Mourant, 1954, p. 158), and has recently recalled how important it was that his assistants were respectable medical practitioners:

The assistants belonged to the tribes concerned, spoke the local languages and were respected for the excellent work they had done to promote health. For example, the assistants vaccinated children, took blood for lab tests and provided antibacterial or antimalarial treatment if required.^24^

Other blood collectors were explicitly coercive. For example, one of Mourant's Brazilian contacts described how the people of one population “objected that they were not sick and thus they did not need any blood examination”. This researcher reported that “a rough clinical examination followed by prescription and even the distribution of some medicine settled the question”.^25^

On the whole however, and beyond Allison's own recollections, details of how blood ownership was negotiated are missing from both published work and private correspondence, giving the impression that blood collections were straightforward. Certainly the extraction of a small quantity of blood did not require the kind of protracted, complex exchanges Warwick Anderson describes in his account of Carlton Gajdusek's acquisition of brains in Papua New Guinea (Anderson, 2008, Anderson, 2013). But recent studies by social anthropologists are of use in conjecturing the possible responses of would-be participants in the 1950s, and suggest that negotiations were far from simple. For East Africa, for example, several works have drawn attention to the narratives of ‘blood-stealing’ and ‘blood-sucking’ associated with ‘vampire stories’ that have been circulating since the beginning of colonial occupation.^26^ Others reflect on multiple local meanings of blood and how those might account for present and past tensions with medical researchers.^27^ The clear message from recent anthropological work, and from the testimonies of participants from the mid-twentieth century to the present, is that encounters were immensely variable, in time and place, between villages and between individuals. Responses must also differ with age and social status, at each stage shaping the constitution of subject populations.

The acquisition of body parts, fluids and tissues is never neutral: we know that strategies for persuading people to donate blood in the British setting depended on propaganda that relied on the fashioning of concepts of altruism and gift giving (Healy, 2006, Whitfield, 2011). Without this kind of apparatus for the careful negotiation of acquisition, blood-taking in remote populations for research purposes can only have been more precarious. Encounters might have involved material exchanges of blood and medicine; they might have been coercive; or blood might have been taken in hospital settings without participants being aware of how it would be used for research.

In summary, collections of blood samples representing ‘populations’ were configured by sampling decisions in the field, the expertise of local assistants, and obligations among kin and community. Further embedded in that data were the, sometimes tacit, assumptions by collectors about who qualified as ‘pure’ members of a particular category. Fieldwork encounters linked the construction of genetic identity to other notions of kinship and belonging.

## 4 From donor records to genetic data

I now want to turn to another kind of collection; not of blood itself, but of the donor cards generated by the transfusion services in Britain. During the Second World War, a new British transfusion service had registered hundreds of thousands of volunteer donors whose records—inscribed with information about blood group—were kept in depots around the country. After the war the new National Blood Transfusion Service (NBTS) expanded its scope, as blood transfusion ceased to be simply an emergency therapy but also began to be used routinely during hospital operations. Mourant became a prominent figure in the NBTS and attended regular meetings with the Regional Transfusion Directors at the Ministry of Health. Using his authority in this setting Mourant brokered the acquisition of donor cards from depots and turned them into data suitable for the geographical mapping of genetic diversity.

In March 1948, British geneticists John Fraser Roberts and Cyril Darlington wrote to the Nuffield Foundation asking for money to support a new research project that would make use of the rich wealth of potential data accumulating in transfusion centres.^28^ Worried that the donor cards were seen as taking up valuable space and might therefore be destroyed, Fraser Roberts and Darlington proposed that the cards could form the basis of a comprehensive survey of the diversity of the whole of Britain.

In the late 1940s and early 1950s Britain was struggling to come to terms with changed relationships within its empire, a demographic crisis, and a severe labour shortage (Paul, 1997). These were challenges to British identity that came to be reflected in the objectives of the new British Ethnography Committee, which the Royal Anthropological Institute established as a “means of promoting the ethnological study of Great Britain”.^29^ In their application to the Nuffield Foundation, Fraser Roberts and Darlington emphasized the importance of Britain for the study of human genetic diversity, owing to its “long and stable history, well authenticated records, high racial diversity and recognised genetic gradients”.^30^ Producing a decisive contrast to the emphasis on racial purity promoted in pre-war Germany, geneticists talking about British diversity tended to emphasize its ‘mixed’ and dynamic composition.

But although Fraser Roberts and Darlington felt that the donor ‘panels’—local lists of volunteers registered with the transfusion service—were potentially ideal resources for mapping the country's genetic diversity, they were careful about what could count as credible genetic data. They worried that people with blood types less useful to the NBTS were resigning disproportionately from panels, and that the records of retired donors were being thrown away owing to lack of space. The panels in many areas were “in danger of being rendered useless for anthropological research by selective removal of cards”.^31^ Fraser Roberts cautioned that unwitting transgressions would threaten the integrity of a region's entire set of records: “If there is any possibility that some of the cards of resigned donors may have been destroyed, the whole record is unusable for anthropological purposes” (Fraser Roberts, 1953, p. 386). So the researchers chose Newcastle-upon-Tyne as their area of study because the transfusion services in the region had an unusually complete set of records. The Nuffield Foundation granted £1000 for the pilot project, and Fraser Roberts decided to base the work at the London School of Hygiene and Tropical Medicine (LSHTM), which was able to provide calculating and punch-card machines.

Fraser Roberts' detailed account of his protocols gives us a rare glimpse of the paper work done to produce genetic data from the records. Because the Newcastle donor records had not been duplicated, Fraser Roberts persuaded the workers there to send their records in batches to the LSHTM and allow the staff in London to copy the “relevant particulars” onto punched cards.^32^ Somewhat surprisingly, given the risk that the donor cards might get lost or damaged, the transfusion director in Newcastle agreed. In copying the cards, Fraser Roberts judged that ‘relevant particulars’ included: (1) a serial number (added to both the card and punched card), (2) whether the donor was on the live panel or resigned panel, (3) the surname of donor, (4) sex and marital status; (5) ABO blood group; and (6) whether the donor was Rhesus positive or negative. Most importantly the researchers at LSHTM gave the donor records a geographical identity. Most people donated blood closer to their place of work than their home, so the researchers could not simply arrange the cards according to transfusion centre; instead they scrutinized the addresses on every record, and used those to locate the donors on an Ordnance Survey map.

**Fig. 2:**
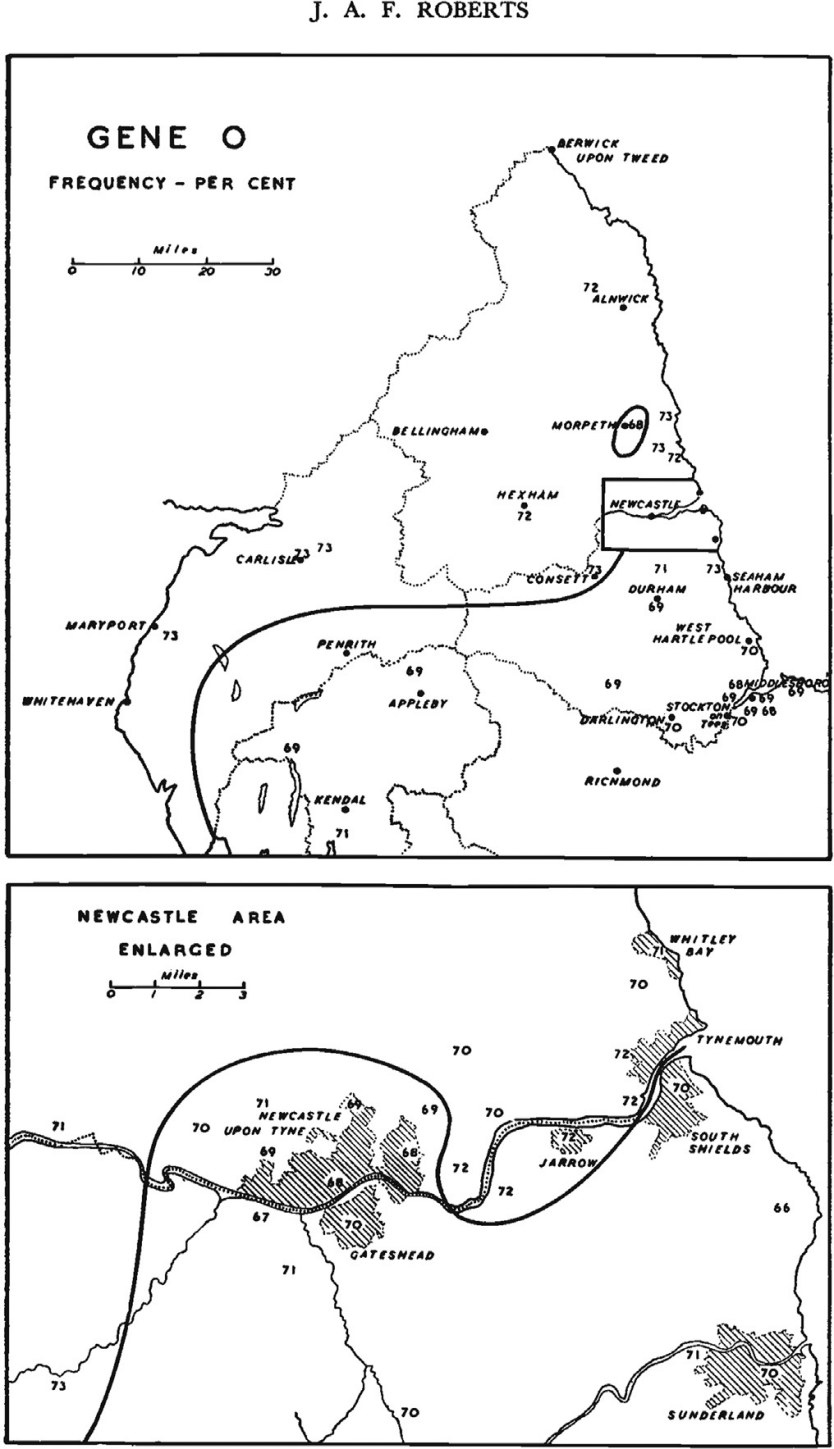
Maps from ‘An Analysis of the ABO Blood-Group Records of the North of England’ by John Fraser Roberts (1953). They show the frequencies of blood-group allele *O* (indicated by the small numbers) in parts of Great Britain around the city of Newcastle. The upper figure shows the whole width of the country, and the lower figure indicates the region around Newcastle itself. Note the dark line apparently indicating a sharp change in the A:O ratio. Pointing to the inscription work used to make this data meaningful, Fraser Roberts explained that a “number of experiments were tried and it soon became clear that it is possible to draw a single line from east to west” (p. 370). Reprinted with permission of Nature Publishing Group, www.nature.com.

A striking difference between the kind of population data produced from collections overseas and those derived from the British donor cards was that for the former the populations under study were generally delimited *before* collection, while for the latter populations were derived *from* the donor records. Starting with the Ordnance Survey map showing the locations of the donors, the researchers aggregated these into groups of 20–70 individuals, keeping separate “individual towns, parts of towns and villages” and adding together “rural areas … where it was practicable.” Crucially, Fraser Roberts also took into account natural and man-made features of landscape, so that “attention was paid to … [the] valleys, roads and railways, that might be expected to facilitate communication”.^33^ Having established these very small population groups, Fraser Roberts sequentially added the groups together to make larger and larger populations, at each step using the chi-squared test to discern whether the new groupings were significantly heterogeneous with regard to blood-group frequency. Eventually, he and his assistants were able to distribute 54,579 donor cards into 321 areas, calculating a blood-group frequency for each of them. Thus the population categories from which Fraser Roberts calculated blood-group frequencies were derived from the internal heterogeneity of the data, and his own judgements about the landscape (Fig. 2).

Fraser Roberts complained that relying on the postal address was “the most difficult and time consuming part” of the study, and he later persuaded the blood transfusion services to “incorporate certain additional points of anthropological interest into the next reprinting of their record cards”.^34^ “Anthropological interest” in this case seems to have meant a coding system for the donor's home address. In his reflections on the practical difficulties of the project, Fraser Roberts explained, “What is required is a code made once and for all, so that any address can be immediately changed into a code number.” He wanted the code to be “hierarchical, so that a count can be made at any desired level of sub-division” (Fraser Roberts, 1953, p. 187). To negotiate this, Fraser Roberts met with members of the Post Office to discuss the coding of postal areas for anthropological research. What we would call ‘postcode’ in Britain today was not systematically introduced until the late 1950s when the Post Office began using electromechanical sorting machines. Before that, large towns and cities in Britain had been divided into several independent areas to facilitate postal sorting, but these were not fine-grained enough for the mapping project. In fact by the time that Fraser Roberts was writing, the Post Office was privately organizing its sorting on the basis of 1700 independent areas and an additional 300 subdivisions of cities, “precisely delineated on maps”. These were not available publically, but Fraser Roberts persuaded the Post Office to make these, and periodic lists of changes to boundaries, available for use in transfusion depots. Fraser Roberts hoped that transfusion centres and donors could be properly disciplined, and suggested that a “directive” might be issued to the Transfusion Centres “pointing out mistakes and omissions and asking them to ensure that addresses are filled in as correctly as possible”.^35^

The pilot study was a great success, resulting in a detailed map of blood-group frequencies distributed across the middle part of Britain (Fig. 2). Building on this, Mourant established the British Blood Group Survey, which went on to collect many more thousands of records from across the country. Why were the Regional Transfusion Directors willing to do this extra work for the sake of an anthropological survey? Not only did it place a further administrative burden on the transfusion service, but also it put records at risk. Part of the answer must be that Mourant had the authority to persuade his colleagues to comply, owing to his regular meetings at the Ministry of Health and his work directing the nationwide distribution of expertise and reagents. At one meeting, Mourant brought Fraser Roberts along to give a lecture on the aims and objectives of the survey and to persuade the transfusion officers to help.^36^ By the time the project was rolled out nationally, Mourant reported that he had been “assured of the friendly cooperation” of his transfusion colleagues.^37^

The British Blood Group Survey ran for more than a decade, and the researchers involved took careful steps to keep their NBTS colleagues interested in the project, giving lectures and presenting maps at meetings and conferences. Nevertheless, it was not self-evident to those working for the transfusion services why the ‘anthropological’ work was important, and Mourant often had to remind transfusion officers to send in their records. Even by 1953, only six of the twelve regional centres were regularly sending in records from new donors, perhaps testifying to the perceived risk to the cards. Mourant reprimanded transfusion directors who were “not yet sending in cards”, and complained to others that “a proportion of the cards submitted by all regions bore incomplete information”.^38^ Eventually, Mourant and Fraser Roberts persuaded the NBTS to introduce a protocol whereby depots would copy the records of newly registered donors at the time of registration, making it easy to send them to the centre.

In short, there were notable differences between the kinds of data collected in Britain and those collected overseas, including whether populations were defined before or after the collections had taken place. Thus the world maps presented in Mourant's book (Fig. 1) brought together populations that were highly variable in scale, resolution and type. Notwithstanding, blood-group collection in all places involved multiple layers of negotiation on the part of researchers and relied on the cooperation of larger-scale institutions, even auxiliary organizations like the British Post Office. In each setting, the scientists judged whether those institutions had appropriate structures and protocols in place to yield useful and credible diversity data.

## 5 The Nuffield Blood Group Centre

In 1951, Mourant wrote to *Nature* to explain that new research on blood group distributions offered “a valuable basis for a genetical interpretation of human diversities”, but he outlined an urgent challenge:

One of the problems is how to collect, assess and make available to anthropologists the vast and rapidly growing mass of data existing in the form of unpublished records and of publications in a very large range of journals.^39^

Mourant's *Nature* letter announced the establishment of a new institution, on the premises of the Royal Anthropological Institute, devoted to the collection, management and analysis of paper records: the Nuffield Blood Group Centre.

The establishment of a blood group clearing house at the Royal Anthropological Institute points to a new agenda for the discipline. After the war, its members had begun discussing how the wartime atrocities of National Socialism had challenged what it meant to study race. In 1946 Herbert Fleure, the new President of the Institute, had used his inaugural speech to announce that it had been “a mistake to divide mankind into groups termed ‘races’”, and to recommend that anthropologists focus their efforts on how “drifts of people in different directions carried ancient characters far and wide”.^40^ Like many of his colleagues, Fleure—who had spent much of his career studying the racial geography and history of Wales—felt that population genetics offered a cogent way of reforming the questions and methods of race science. In line with arguments developed in the 1930s, and soon to be deployed publically by UNESCO in their campaign against racial prejudice, Fleure declared that “it is Mendel who has helped us to see that we are bundles of heritages which get re-sorted and recombined for every birth”, and he urged anthropologists to “welcome increased co-operation from researchers in genetics” (Fleure, 1946, p. 2). To promote such collaborations, Fleure offered his support to the establishment of a Blood Group Committee at the Royal Anthropological Institute, which would evaluate the scope and direction of research on genetic variation.

In 1951, Fleure chaired the committee's first public event: a “special meeting” for “anthropologists, serologists and geneticists to survey the functions and need of blood group studies in anthropology”. Held in the Eugenics Theatre at University College London, the meeting began with research papers from “practically all the leading workers” on blood group genetics—including Fisher, Mourant, Darlington and Fraser Roberts—and was followed by a discussion devoted to garnering wider opinion on the establishment of a new “Reference Centre for Results”. This new centre, it was decided, would “correlate, tabulate and report on all blood group research having a bearing on anthropology”, and would formalize some of the work that Mourant was doing to foster the “interchange of information” between researchers.^41^ It would also offer advice on how to organize fieldwork, how to analyse data, and how to publish results.^42^

The centre was established in 1952 with Mourant overseeing its work, a role he took on alongside his continued responsibilities at the Blood Group Reference Laboratory. The Nuffield Foundation provided £14,000 for the centre's first five years. Working in a small cottage at the back of the Royal Anthropological Institute building in Bedford Square, clerical assistant Kasimiera Domaniewska-Sobczak and librarian J. W. Wasung classified offprints on blood group distributions in a card index, and organized and catalogued incoming data “in preparation for computation”. Statistician Ada Kopeć used a “Monroe calculating machine” to carry out this “computation”.^43^ Published and unpublished data arrived at the centre from Mourant's serological network of blood banks, transfusion depots, colonial hospitals, public health centres, and university departments. Correspondents were frequently carrying out WHO (or occasionally military or missionary) public health work, but were also interested enough in new research on the diversity of blood groups to do small-scale research projects on the side. Mourant embedded his requests for data in letters that discussed the provision of different kinds of antisera, serological techniques, and the blood-grouping services that the BGRL could offer. Testifying to Mourant's dependency on his WHO connections, his 1954 maps of the Rhesus blood-group alleles show the Soviet Union as a white space; at this time the Soviet Union was not a member of the WHO and so Mourant had no Rhesus data from there (Fig. 1).^44^

**Fig. 3:**
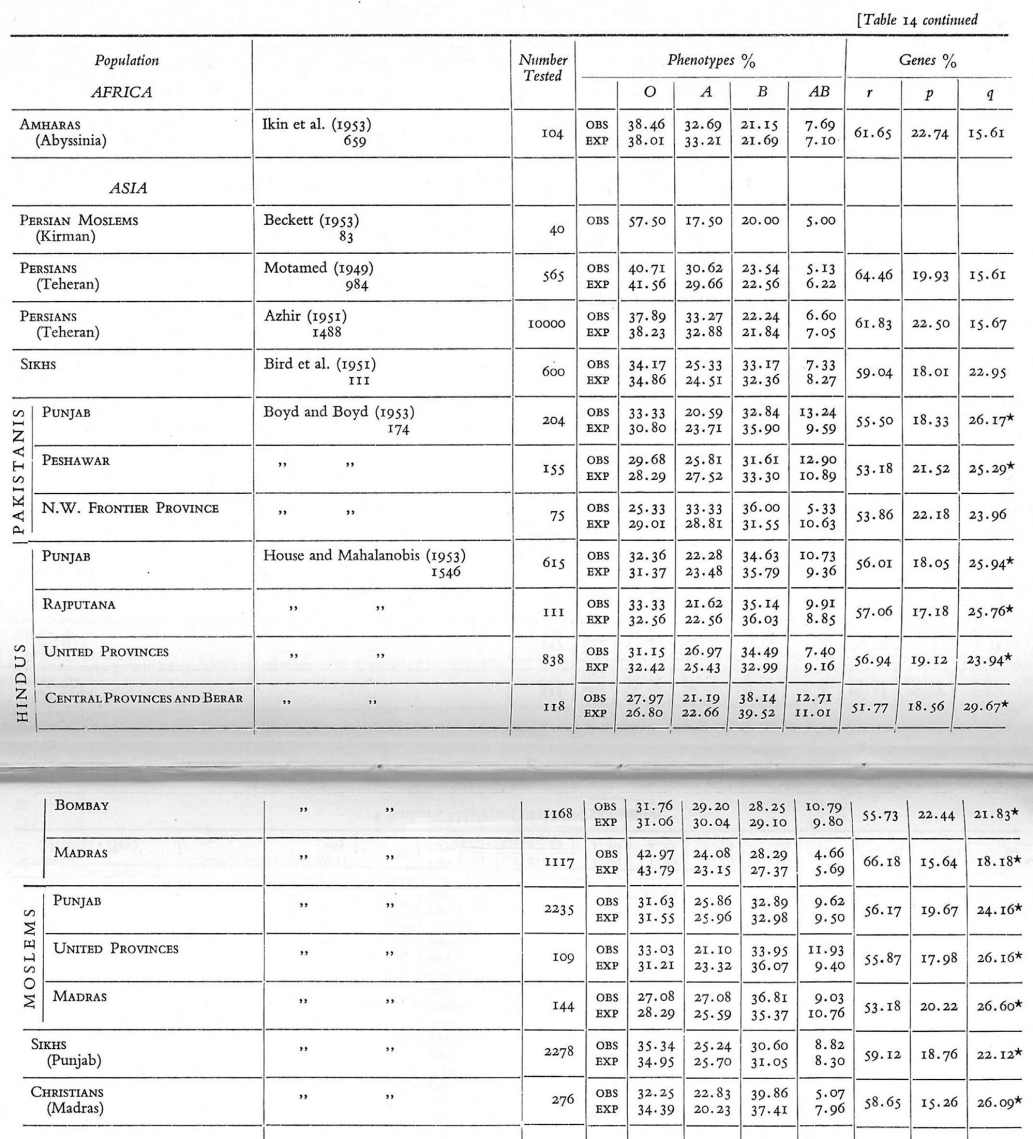
Section of table 14 from *Distribution of the Human Blood Groups* (1954) showing the frequencies of the ABO groups. The table displays (from right to left) population (national, religious, racial, geographical) categories, citations to the papers where the data is from, the number of people tested, observed and expected proportions of the different ABO blood groups (phenotypes), and proportions of the ABO alleles (*r*, *p*, *q*). See Fig. 1 for permissions information.

**Fig. 4:**
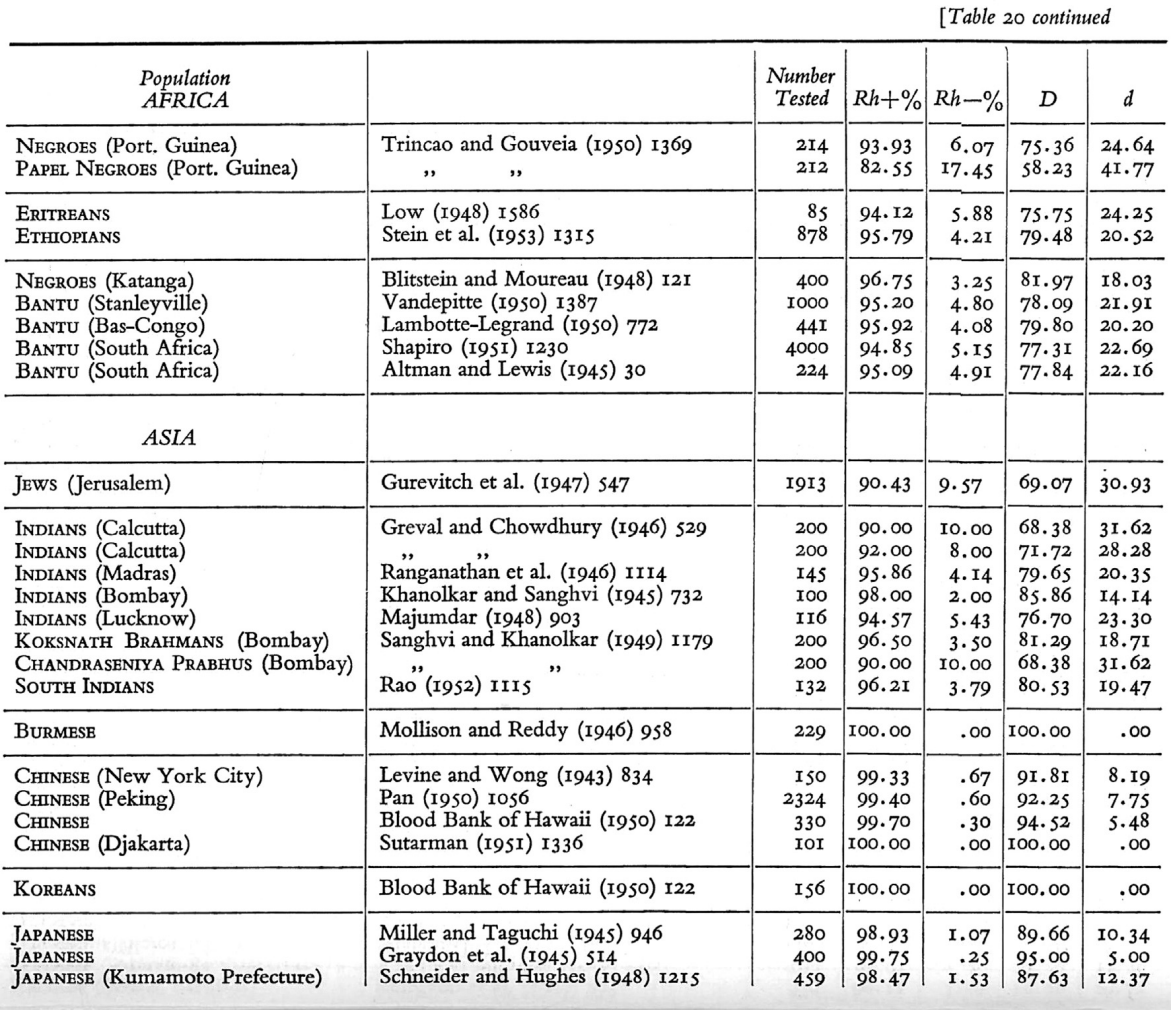
Section of table 20 from *Distribution of the Human Blood Groups* (1954) showing the frequencies of the Rhesus blood groups. The table displays (from right to left) population (national, religious, racial, geographical) categories, citations to the papers where the data is from, the number of people tested, the percentage of people tested as Rhesus-positive and Rhesus-negative, and proportions of the *D* and *d* alleles. See Fig. 1 for permissions information.

Together, Kopeć, Domaniewska-Sobczak, and Wasung classified the data on blood-group distributions, assessed it “statistically” and made “the results available to anthropologists and other research workers”, leading to the publication of *The Distribution of the Human Blood Groups*.^45^ The 40 large tables in the back of the book offer some insights into the work that went on there (Fig. 3, Fig. 4). For each population, the tables present the numbers of blood samples tested and the percentage of individuals carrying each blood group (under the heading ‘phenotypes’). When blood-group results arrived at the centre, clerk Domaniewska-Sobczak filed the results according to the cultural, historical, geographical or political categories, presumably in a similar way to the tables, where population groups perceived to have close relationships are clustered together. In the tables, larger geopolitical regions (e.g. ‘Africa’ or ‘Asia’) are subdivided by nation, religion or caste (‘Jews’, ‘Koksnath Brahmans’), and sometimes further divided into smaller regions (e.g. ‘Lucknow’, ‘Bombay’ etc.), or even according to the researcher's perception of what percentage ‘Indian’ the research subjects were.^46^ Horizontal lines delineating the rows in some instances denote national boundaries and sometimes more opaque divisions. Just as with Mourant's Basque collections, geographical category names did not necessarily indicate where the blood was collected; someone qualifying as ‘Chinese’ evidently could have had their blood collected in the United States (Fig. 4).

The tables also give some indication of the statistical analysis carried out by Kopeć. The rows on the furthest right quote blood-group results not in terms of the percentage of individuals with a given blood group, but rather the percentage frequency of *alleles* in that population (in Fig. 3, the ABO alleles are denoted *r*, *p* and *q*; in Fig. 4, the Rh alleles are called *D* and *d*).^47^ Quite routine in population genetics, quoting allele frequencies rather than blood-group frequencies often had the effect of amplifying apparent differences between populations.^48^ For example, owing to the dominance behaviour of different alleles, 50% of a given population might be blood-group O, but 71% carry the *O* allele.

The tables also testify to an important method used to verify the internal consistency of the data, which used a general principle of population genetics—the Hardy–Weinberg equilibrium. Each frequency column in Fig. 3 contains two rows—an upper one for ‘observed’ and a lower one for ‘expected’ values. The Hardy–Weinberg equilibrium states that if a population is large and, in general, randomly mating, then for gene variants at any one locus the proportion of heterozygotes to homozygotes stands in fixed relation to one another. So where the table shows ‘expected’ values, these are ‘values expected given Hardy–Weinberg equilibrium’. Any significant deviation of the observed from these might, to a population geneticist, indicate that the requirements for equilibrium were not fulfilled (large population etc.) or that the locus was under selection. The most likely explanation, however, was that the grouping tests themselves had been inaccurate, perhaps owing to ineffectual reagents.^49^ So in a section of his book on Tibet, Mourant could write: “Tennant's observations on 187 Tibetans must be viewed with considerable caution since he found 24.1 per cent of AB's [*sic*], a very much higher frequency than would be expected on the basis of genetical equilibrium” (Mourant, 1954, p. 117). Thus this population-genetic technique served as a means for assessing the credibility of the data.

Mourant's other defence against spurious results was his network of correspondents. Mourant's own staff at the BGRL had taught blood-grouping techniques to many of his contacts around the world, and he had clear views about the competence of different laboratories. But the most important way that Mourant guaranteed the credibility of the data was simply by accumulating big numbers. He reasoned that in the end errors would be swamped by the vast quantities of data that would be collected in the future. As one of his reviewers pointed out, more or less approvingly:

Mourant tends to give those who report the data the benefit of any doubts which exist, apparently believing that tolerance is likely to produce more data to which time will apply the corrective. (Dunn, 1955)

Population genetics was a statistical science and Mourant judged that this approach could well be tolerated by the standards of the field.

## 6 *The Distribution of the Human Blood Groups* (1954)

Mourant's *magnum opus* was a 440-page cloth-bound book containing 240 pages of text; it was the third of three books published by Blackwells devoted to new research on blood groups. The first had been *Blood Groups and Transfusion*, written by Patrick Mollison, who was a transfusion specialist and close colleague of Mourant's in charge of another Medical Research Council laboratory in London. The second was the definitive textbook on blood-group inheritance: *Blood Groups in Man*, by Robert Race and Ruth Sanger. Robert Race ran the Lister Institute's Blood Group Research Laboratory, which was the adjacent ‘sister’ lab to Mourant's BGRL.^50^ The foreword to *The Distribution of the Human Blood Groups* was by Herbert Fleure, whose support of the book testifies to the perceived significance of blood-group studies for addressing questions about race. Fleure wrote that despite the need to interpret the data cautiously, “the study of individual characteristics and of their relations with one another, especially in their geographical distribution among percentages of different populations, is the most hopeful road towards understanding human diversity”.^51^ Focussing on *Distribution* I want to end by making a few remarks about what Mourant decided to reveal and conceal about his collecting practices, and how those choices functioned.

**Fig. 5:**
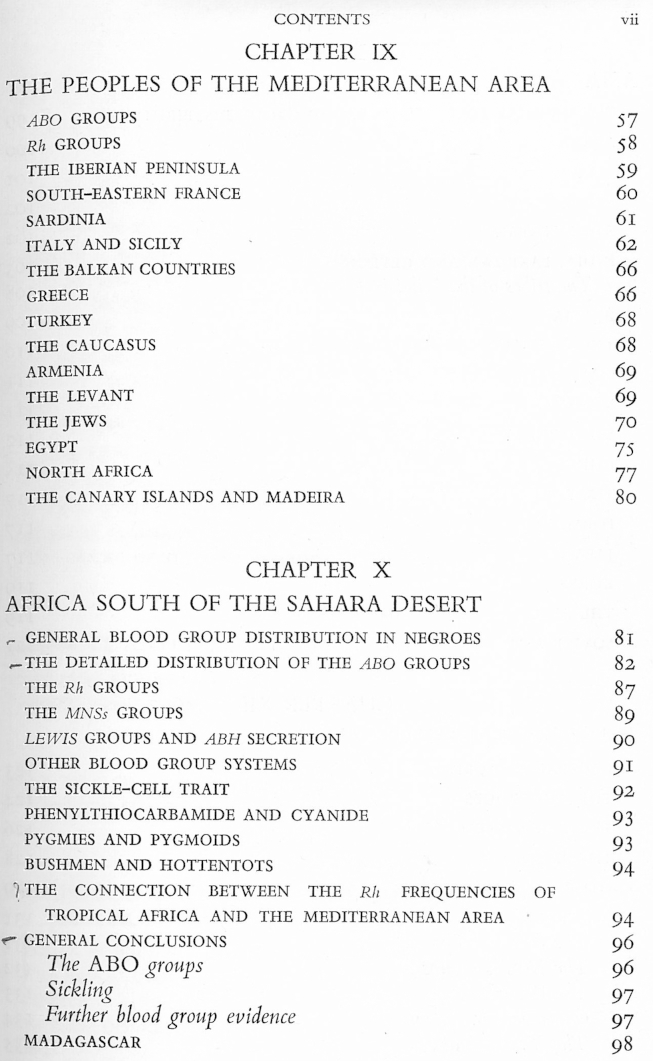
A section of the contents pages of *The Distribution of the Human Blood Groups* (1954) giving a sense of the nested chapter organization of Mourant's book in relation to geographical, national, religious and racial categories. See Fig. 1 for permissions information.

Mourant organized many of the chapters of his book according to geopolitical, religious and ‘tribal’ groupings, similar to those described in the tables (Fig. 5). These chapters consisted of long textual descriptions of the blood-group frequency distributions, alongside information about the geography, anthropology, history, and languages of the groups under study. To give a lengthy example:

The Ainu differ greatly in physical characters from the Japanese and all other eastern Asiatic peoples. They live in Hokkaido and in the Soviet island of Sakhalien. According to some authorities an Ainu strain is detectable in the Japanese of other parts of Japan. Owing to the Caucasoid appearance of the Ainu there has been much speculation as to their connections with the peoples of Europe and the Aborigines of Australia. … From the data quoted by Boyd it is seen that the overall gene frequencies both in Hokkaido and in Sakhalien are near 23 per cent for *A* and 28 per cent for *B*, and these figures probably give as good an estimate as possible for the Ainu as a whole. For Hokkaido, Simmons et al find *A* gene frequencies of 26 to 31 per cent, and *B* gene frequencies of 9 to 36 per cent. In general it may thus be said that the Ainu have less *A* but more *B* than the Japanese. (Mourant, 1954, p. 119)

Despite the range of information that Mourant marshalled in these kinds of descriptions (geographical and political in this quotation), he presented not a single reference to any paper that dealt with the production of historical, linguistic or anthropological knowledge. By leaving out the sources for his information about the populations under study, Mourant presented their identities as taken-for-granted. Moreover, Mourant rarely mentioned how individuals were recruited as research subjects, and where he did, it was almost always with reference to a ‘local’ expert, e.g. de Barandiarán's ‘expert guidance’ on ‘pure Basques’, and Yturri's skill at identifying Basques with ‘intact lineages’. Later in the 1950s, standards for sampling shifted, and blood collectors began recruiting linguists and social anthropologists to give advice on whose blood should be collected.^52^ But in the early 1950s Mourant and his colleagues simply presented ‘local’ assistants as having authentic and reliable knowledge about the qualification of individuals to subject populations. By introducing these ‘assistant’ characters in published reports the researchers obscured further details about how those choices were made, and helped to characterize the relationships between research subjects as self-evidently credible. Through these strategies, Mourant stabilized assumptions about which populations were genetically interesting.

Mourant's presentation of blood-group data—and the fact that he and other researchers rarely concluded anything dramatically new about the populations they studied—suggests that blood groups were not living up to the lofty ambitions made for them. However, we might see the strategies that Mourant used to present blood-group results as serving to *calibrate* blood-group genetic data in relation to existing knowledge.^53^ Lists of blood-group gene frequencies would never have been enough; to make genetic data say anything meaningful about human groups they had to be aligned with contemporary racial, historical, and geographical knowledge.

Continuing the metaphor of calibration, Mourant even referred to his book as an “instrument for research”, a characterization that was echoed by Joseph Birdsell who called it an “important and finely polished research tool” (Birdsell, 1956). “Genetical anthropology,” as Mourant put it, was a young science, and the “study of mixed populations” was “only just beginning”:

[I]n the present stage of genetical anthropology the main task is the most complete possible genetical analysis of the parent populations, for only with the help of this knowledge can the history of their descendants be deduced. (Mourant, 1954, p. 148)

Apparently, when enough data had been collected on the “parent” populations of the world, blood-group frequencies would be a fit instrument for probing more subtly mixed populations.

## 7 Conclusion

Mourant's work offers glimpses of the kinds of practices used to produce blood-group genetic data, from the bodies that yielded blood to the presentation of population data on the printed page. ‘Populations’ were not simply given, but were produced during the sequence of practices for their collection, collections that varied according to the institutions and people recruited to them. The map shown in Fig. 1 represents population data that varied in scale, resolution and type, and that were profoundly shaped by colonial governance, by the World Health Organization, by auxiliary institutions such as the British Post Office, as well as relationships between collectors and subjects and by the labour and expertise of assistants and mediators.

The construction of blood groups as devices for probing human biological difference relied on notions of kinship, racial and national identity, multiple historical traditions, and questions and practices imported from diverse disciplines. In other words, the varied work that went into making populations into biologically relevant entities was not simply or self-evidently ‘biological’. Side-stepping the question of whether this recourse to ‘cultural’ knowledge was disingenuous or naïve, this work made blood groups look like they could yield convincing knowledge about human life and its history. Notwithstanding Mourant's claims about blood groups being “objective criteria” for studying race, we might view his book's purpose as not *yet* to probe the origins of ancient peoples, but to calibrate a new tool: blood-group genetic data. More broadly, Mourant's project offered a cogent way of rendering genetics ‘human’: aligning blood-group frequencies with human populations gave the data the potential to reveal new knowledge about human difference, ancestry and history, even if Mourant's conclusions were deferred. On the one hand this is a story of the way in which human difference was made genetic; on the other it is about how genetic data were made human.

## Footnotes

1 For Germany see Mazumdar (1990) and Boaz (2012); for Hungary and Romania see Turda (2007); for valuable overviews see Schneider, 1995, Schneider, 1996.

2 This estimate comes from Schneider (1996).

3 For sustained accounts of the procurement of body parts for scientific research see Lindee (1998) and Anderson (2008).

4 Wellcome Library, PP/AEM/C.1, Mourant Papers, Mourant, “Progress Report 1949–50 of the Blood Group Reference Laboratory,” 1950.

5 In addition to those works cited above, see, for example, Marks, 1996, Kirsh, 2003 and Lipphardt (2010).

6 For an exemplary overview of the production of racial and national meanings through blood group maps, see Gannett & Griesemer (2004).

7 As this paper focuses on collection practices, it does not deal with the procedures used to test blood groups. For more on blood-grouping protocols, and on the production of blood groups as inscriptions, see Bangham, 2013a, Bangham, 2013b.

8 ‘Polymorphism’ is a term that simply refers to a gene that has different variants in a population.

9 Quotation from a British anthropologist named (confusingly) Geoffrey Morant (1929, p. 67).

10 Araquistain (1945, p. 33) and de Barandiarán (1946, p. 96).

11 See Taylor (1921, p. 83) and de Barandiarán (1946, p. 96).

12 Allison has been extremely generous with his recollections, via email, of his early collecting expeditions. Allison later became well known for his suggestion that people carrying the sickle-cell anaemia trait were more resistant to malaria. His 1949 expedition to Kenya was where he first formulated this idea, although at that time the sickling tests were a sideline to his work surveying blood-group frequencies.

13 “Letter to Oxford University Registrar,” February 14, 1949, Oxford University Exploration Club archive.

14 ‘Lapps’ was the term used in the 1950s for Sami people, a Finno-Ugric group consisting of different communities living in far northern Sweden, Norway, Finland and the Kola Peninsula of Russia. The Sami are protected under the Indigenous and Tribal Peoples Convention.

15 Most of the remaining money came from the University of Oxford, the Oxford colleges, and expedition participants, but the organizers also elicited a small proportion from the British Museum and from the Royal Geographical Society, which routinely contributed money to Oxford University expeditions.

16 For work on disease and blood groups at this time, see for example Race & Sanger (1950). For the general aims and preferences of the Colonial Research Committee, see Clarke, esp. pp. 470–479 (2007).

17 A. C. Allison (personal communication, March 12, 2012).

18 Technical instructions comprised much of Mourant's contribution to the 1951 edition of *Notes and Queries in Anthropology*, which was the Royal Anthropological Institute's guide to fieldwork practices (Royal Anthropological Institute of Great Britain and Ireland, 1951, p. 16).

19 These were Mourant's own inverted commas, suggesting that by the mid-1950s he hesitated over the term ‘purity’ (Mourant, 1954, p. 41).

20 Both quotations come from A. C. Allison (personal communication, March 12, 2012).

21 W. C. Laughlin, “To Mourant,” April 3, 1951, PP/AEM/K.99, Wellcome Library.

22 (Royal Anthropological Institute of Great Britain and Ireland, 1951, p. 19).

23 A. C. Allison (personal communication, March 12, 2012).

24 Although Allison relied on his colleagues for help with translation, he was fluent in Kiswahili and so recalls being able to talk to chiefs himself: A. C. Allison (personal communication, March 12, 2012). For reflections on the history of biomedical research in East Africa in the 1950s and the complex dynamics of recruitment and consent, see Graboyes (2010).

25 The paper by E. M. da Silva (1949) was one of several blood-grouping articles published alongside Mourant's Basque publication in a single issue of *American Journal of Physical Anthropology*.

26 For other mentions of the term ‘vampire’, both descriptively and critically, in relation to biomedical research, see for example e.g. Reardon (2004, p. 2) and Radin (2012, p. 191). In a sustained account of blood-stealing rumours, Luise White talks of a “transcolonial movement of vampire accusations” that expressed local issues and concerns about colonization (White, 2000, p. 206).

27 Wenzel Geissler writes about the power that Luo people (one of the tribes in Kenya mentioned by Allison) attribute to blood. Geissler also reviews the diverse experiences of medical officers in Uganda and Kenya from the early twentieth century. One reported having been sent presents to extend his research in neighbouring villages; another recalled violence by both medical officers and villages (Geissler, 2005).

28 Roberts had became interested in British blood-group diversity while volunteering at a wartime transfusion centre in Bath (Polani, 1992). Darlington was already an eminent plant cytogeneticist, enthusiastic eugenicist and public advocate for genetics, who took an interest in blood-group distributions in the 1940s (Harman, 2004).

29 The committee later hosted a lecture on the British blood-group survey. Royal Anthropological Institute archive, 59/10/1.

30 C. D. Darlington, “Letter to General Bullen Smith (of the Nuffield Foundation),” March 31, 1948, A91/2/7, Royal Anthropological Institute.

31 C. D. Darlington, “A Proposed ABO Rh Blood Group Survey. SA/BGU/91/2/6,” estimated 1948, SA/BGU/91/2/6.

32 Fraser Roberts (1953, p. 362).

33 Fraser Roberts, “An Analysis of the ABO Blood-Group Records of the North of England,” 365.

34 Fraser Roberts (1953, p. 363); Fraser Roberts, “The Coding of Postal Addresses,” October 15, 1953, A91/2/16, Royal Anthropological Institute.

35 Fraser Roberts, “The Coding of Postal Addresses,” October 15, 1953, A91/2/16, Royal Anthropological Institute

36 William d'A Maycock, “To R. H. Malone, Sheffield Regional Transfusion Laboratory,” April 5, 1951, BN 13/31, National Archives.

37 “Meetings of Regional Transfusion Directors: Minutes,” October 3, 1951, BN 13/31, National Archives; A. E. Mourant, “Proposal from Mourant to Nuffield Foundation,” June 6, 1951, PP/AEM/C.9.

38 “Minutes, Regional Transfusion Officers Meeting, Ministry of Health,” September 30, 1953, BN 13/31, National Archives.

39 Mourant, “Blood Groups and Anthropology: Draft” 1.

40 Fleure's lecture dealt with the state and future of the Institute, and with “the scope of anthropology”, especially its relation to archaeology, prehistory and the improvement of human welfare. Quotations from Fleure (1946, p. 2).

41 Mourant, “Draft Press Release to ‘Eugenics Review.’” As described in the essay by Veronika Lipphardt (in this issue) the early 1950s saw the establishment of two other centres devoted to the study of human genetic diversity data: Leslie C. Dunn's Institute of Human Variation at Colombia University in New York and L. D. Sanghvi the Laboratory for Studies in Human Variation in Bombay.

42 A. E. Mourant, “Letter for the Nuffield Foundation, draft,” May 30, 1951, A91/2/1, Royal Anthropological Institute.

43 A. E. Mourant, “Nuffield Blood Group Centre, Report to Council,” April 23, 1952, A91/2/14, Royal Anthropological Institute.

44 The Soviet Union withdrew from the WHO in 1949 and returned in the mid 1950s.

45 A. E. Mourant, “Draft Press Release to ‘Eugenics Review,’” 1952, A91/2/2, Royal Anthropological Institute.

46 Table 14 in Mourant's book separately lists “Indians (British Columbia), Chippewa Indians (Full Blood), Chippewa Indians (>¾ Indian), Chippewa Indians (<¾ Indian)”.

47 The calculations used to make the transformation from blood-group frequency to allele frequency were different for each of the blood group systems, and Mourant devoted a whole chapter in *Distribution* to ‘Gene Frequency Calculations’.

48 On the effect of increasing differences, Nelly Oudshoorn makes this observation in relation to the representation of women and menstrual cycles in pill trials (Oudshoorn, 1994).

49 During the war Mourant's colleague Fisher had applied this protocol to blood-transfusion records sent to him by depots; by the postwar period it had been made into a routine step in the protocol for producing genetic data; see Bangham (2013b).

50 Mourant and Robert Race often shared sera, contacts, and specimens and the two labs were sometimes seen as working together as a unit. The complementary activities of the two labs are dealt with by Bangham (2013a).

51 See ‘Foreword’ by Fleure in A.E. Mourant, *The Distribution of the Human Blood Groups* (Oxford: Blackwell Scientific Publications, 1954), xix.

52 See, for example, the criticism by Bruce Chown (1957) in response to the paper by Kraus & White (1956); the contributions of linguists and cultural anthropologists to biomedical research in the 1960s is discussed by Edna Suárez-Díaz (in this issue).

53 I take the term ‘calibration’ in this sense from Nick Jardine, who uses it to describe how a new theory might successfully substitute for an old one; see Jardine (1991). For another specific application of the term, see Hacking (1995, pp. 96--112). Drawing on these, I use ‘calibration’ to point to the work done to successfully align the methods and judgements offered by blood-group genetics with those previously produced through the study of physical anthropology, geography and history.
